# Naltrexone-Associated Non-ST-Elevated Myocardial Infarction

**DOI:** 10.7759/cureus.11198

**Published:** 2020-10-27

**Authors:** James C Gubitosa, Toby Terwillliger, Adanna Ukazu, Emily Gordon

**Affiliations:** 1 Medicine, University Hospital - Rutgers New Jersey Medical School, Newark, USA; 2 Medicine-Pediatrics, University Hospital - Rutgers New Jersey Medical School, Newark, USA; 3 Addiction Medicine, University Hospital - Rutgers New Jersey Medical School, Newark, USA

**Keywords:** non-st segment elevation myocardial infarction (nstemi), naltrexone, opioid use disorders, ascvd, medication-assisted treatment, medication for opioid use disorder (moud), opioid agonist therapy (oat), cardiac troponin, demand ischemia, opioid withdrawal

## Abstract

Medications for opioid use disorder (MOUD) and opioid agonist therapy (OAT) are the mainstays of treatment in opioid use disorder. Significant caution is encouraged upon initiation to reduce the precipitation of opioid withdrawal. Cardiac events in the setting of opioid withdrawal are rare and incompletely understood.

A 46-year-old woman with a history of opioid-use disorder, hypertension, hyperlipidemia, diabetes, tobacco-use disorder, and rheumatoid arthritis presented with nausea, vomiting, and lightheadedness after taking naltrexone following buprenorphine. She was found to be hypertensive and tachycardic in the emergency department, with a troponin of 0.38 ng/mL (reference: 0.00-0.30 ng/mL) and an electrocardiogram (ECG) without ST or T-wave changes. She was admitted for a non-ST-elevation myocardial infarction (NSTEMI) and hypertensive emergency in the setting of opioid withdrawal. Her blood pressure was controlled, and she received full-dose aspirin and high intensity atorvastatin. Afterwards she was started on a modified OAT regimen of buprenorphine 8 mg daily. Her cardiac enzymes down-trended and her condition became stable after which she was discharged home.

Cardiac events are an uncommon yet lethal occurrence in opioid withdrawal. The likely etiology of NSTEMI in our patient was demand ischemia induced by opioid withdrawal, augmented by her various other cardiac risk factors. Practitioners should be aware of these possible adverse events, especially in those with preexisting cardiac disease. Meticulous efforts should be made to instruct patients as to the proper dosing schedule when initiating opioid therapy, and when initiating MOUD/OAT in order to prevent poor outcomes.

## Introduction

Medications for opioid use disorder (MOUD) and opioid agonist therapy (OAT) represent efficacious and evidence-based treatment regimens for opioid use disorder. These therapies stabilize brain chemistry and reduce cravings allowing return to full functionality without the adverse health effects of prescription opioids or heroin. Commonly used medications include buprenorphine, buprenorphine-naloxone, methadone, and naltrexone. MOUD/OAT must be initiated with care and counseling to avoid inadvertently precipitating opioid withdrawal. Opioid withdrawal can manifest as lacrimation, salivation, yawning, piloerection, nausea, abdominal pain, diarrhea, myalgias, anxiety, restlessness, or sweating [1]. While exquisitely uncomfortable, opioid withdrawal is not considered to be life-threatening or to have long-term adverse health effects. Cardiac stressors such as exercise, and emotional stress have been associated with cardiac ischemia in those with underlying coronary artery disease (CAD). However, no evidence specifically linking opioid withdrawal with coronary events exists in the literature [2]. Our review uncovered only one case report in which a 64-year-old man with pre-existing CAD presented with acute myocardial infarction in the setting of opioid withdrawal [3]. We are not aware of any reported cases of opioid withdrawal-induced ischemia in those without premorbid coronary artery disease. In the current study, we describe a patient on MOUD with no documented history of coronary disease, who presents with an incident cardiac event in the setting of naltrexone-induced acute opioid withdrawal.

## Case presentation

A 46-year-old female with a history of opioid-use disorder, hypertension, hyperlipidemia, diabetes, tobacco-use disorder, and rheumatoid arthritis presented to the emergency department (ED) with nausea, vomiting, and lightheadedness after taking naltrexone. The patient had a longstanding history of opioid abuse, including prescription tramadol and intranasal heroin, and attempted to quit multiple times. She successfully completed an inpatient drug rehabilitation program two weeks prior to the current presentation and was sent home with a one-week buprenorphine taper, with instructions to begin naltrexone 50 mg daily after completion of the taper. However, the patient had prior prescriptions of buprenorphine in the home from prior periods of attempted abstinence, which she continued to take up until the day prior to presentation. She had not taken naltrexone up until the day prior to admission. On the day prior to admission, the patient took an AM and PM dose of buprenorphine 1 mg, followed by a single evening dose of naltrexone 25 mg. Hours later, the patient began to experience severe nausea, vomiting, and abdominal pain, for which she presented to the ED.

**Figure 1:**
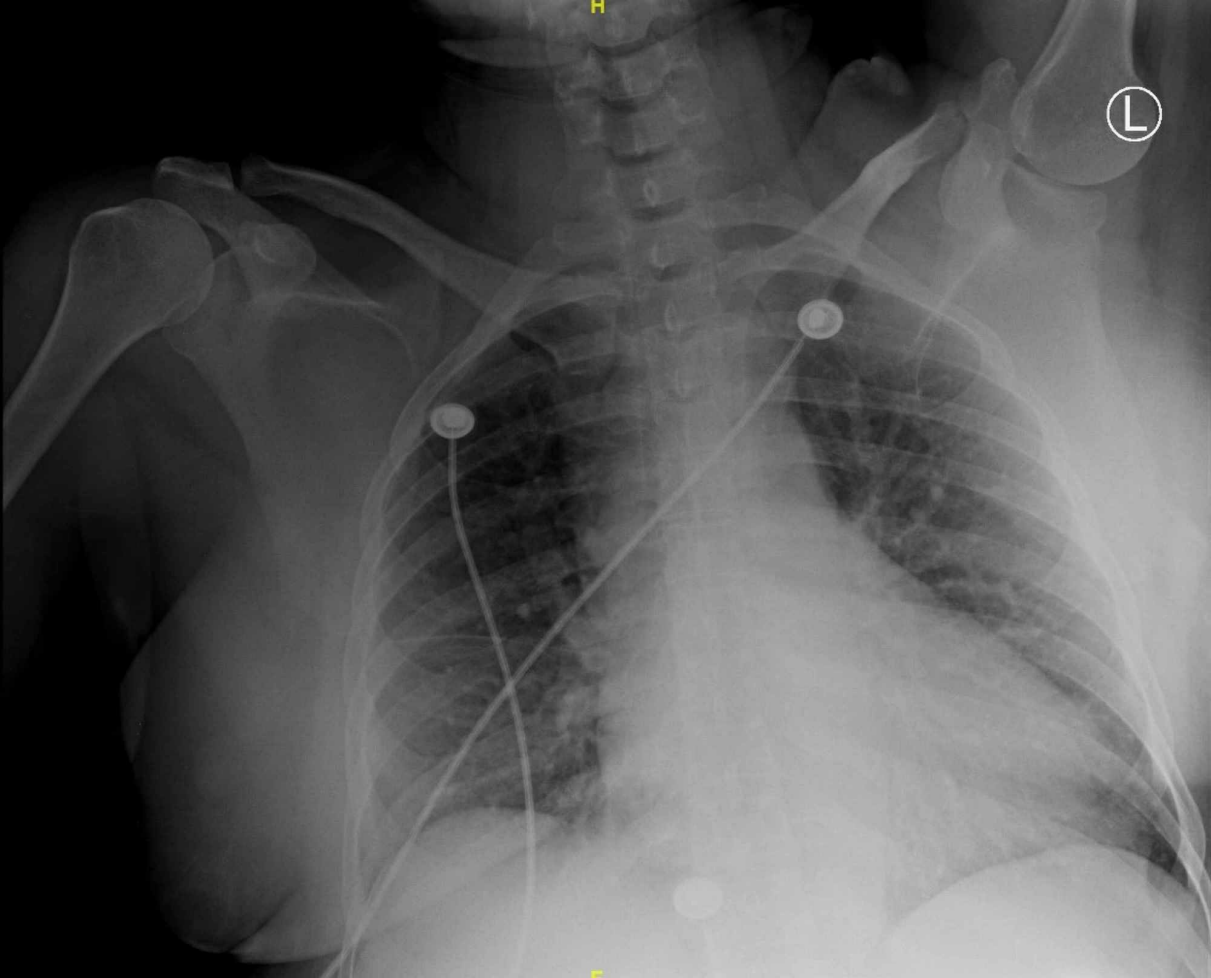
Anteroposterior X-Ray of the Chest Demonstrating clear bilateral lung fields with overlying breast tissue opacities and a normal-sized cardiac silhouette.

**Figure 2:**
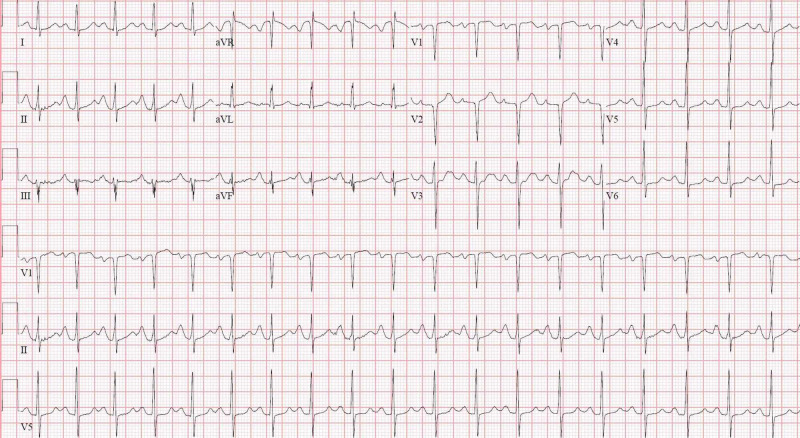
Twelve-lead electrocardiogram Demonstrating sinus tachycardia with evidence of right atrial enlargement.

In the ED, vitals were significant for a blood pressure of 188/110 mmHg and heart rate of 122 beats per minute (bpm). Additionally she had a troponin of 0.38 ng/mL (reference: 0.0-0.30 ng/mL), a normal chest X-ray (Figure 1) and an electrocardiogram (ECG) showing right atrial enlargement (RAE) and sinus tachycardia, without any ST or T-wave changes (Figure 2). Intravenous labetalol 10 mg was given, and the patient was restarted on her home dose of lisinopril 10 mg, with improvement of her blood pressure to 119/78 mmHg. Aspirin 325 mg and atorvastatin 80 mg were subsequently given. The Clinical Opioid Withdrawal Scale (COWS) was calculated to be 17. Sublingual buprenorphine 2 mg was given without improvement. The patient was then admitted for management of hypertensive emergency and non-ST-elevated myocardial infarction (NSTEMI) in the setting of opioid withdrawal.

Addiction medicine was consulted, and the patient was started on 8 mg of buprenorphine daily with improvement of her COWS to <10. Her cardiac enzymes decreased to normal limits by the following day and she felt significantly better. No further ECG changes were observed. She was subsequently discharged home in stable condition on buprenorphine-naloxone 8-2 mg sublingually twice daily, as well as follow-up visits with cardiology and addiction medicine.

## Discussion

The differential etiology of an NSTEMI as it pertains to naltrexone use is broad. Given our patient’s significant cardiac risk factors, demand ischemia in the setting of subclinical atherosclerotic cardiovascular disease is likely. However, one should also consider the possibility of naltrexone-induced cardiotoxicity, arrhythmia, and other coronary pathology.

Naltrexone is a pure competitive opioid antagonist commonly used in MOUD for opioid-use disorder. It is similar to the opioid pain medication, oxymorphone, albeit with a tertiary amine methyl group replaced by methylcyclopropane [4]. This modification allows it, and its active metabolite 6β-naltrexol to reversibly inhibit the μ, κ, and δ-opioid receptors [5]. It has been shown to precipitate rapid withdrawal in patients actively taking opioids [6], and thus its concomitant use with OAT options such as buprenorphine or methadone is contraindicated. When our patient took 25 mg of naltrexone shortly after her second daily dose of 1 mg buprenorphine, withdrawal was predictably initiated and an adverse cardiac event in the form of an NSTEMI occurred. Upon review of the literature, no documented evidence of direct cardiac toxicity from naltrexone or naltrexone-induced arrhythmia exists. Oral naltrexone tablets contain several inactive metabolites including colloidal silicone dioxide, methylcellulose, and magnesium stearate - all of which are not known to cause cardiac complications. Other common side effects of naltrexone include diarrhea and abdominal cramping [4].

A myocardial infarction linked to naltrexone administration has only been cited in one prior case, in a patient with documented CAD [3]. The most likely etiology of this event in our patient is that of demand ischemia in the setting of opioid withdrawal. Our patient had numerous cardiac risk factors including uncontrolled hypertension, hyperlipidemia, diabetes, and active tobacco use. Her 10-year atherosclerotic cardiovascular disease (ASCVD) risk was calculated to be 53% via the Pooled Cohort Equation despite her young age of 46. Although she did not receive cardiac catheterization nor cardiac stress-testing while inpatient, it is plausible to consider the existence of existing ASCVD and potentially compromised coronary arteries. Her hypertensive emergency (with blood pressure 188/110 mmHg) on presentation would have greatly increased her cardiac afterload. This coupled with her tachycardia (122 bpm) would have led to increased myocardial oxygen consumption and likely precipitate myocardial damage resulting in her observed troponinemia and diagnosis of NSTEMI.

Research over past decades has suggested that opioids inherently exhibit some form of cardioprotective effect [7]. The κ-opioid receptor in rat hearts was studied by Rong et al. and found to be linked to a reduction in necrosis and apoptosis following cardiac ischemia and reperfusion (I/R) when a pure κ-agonist was given [8]. Methadone and morphine and their effects on the κ and δ-opioid receptors within rat cardiomyocytes were studied by Gross et al. and showed similar reductions in I/R injury [9]. Maslov et al. observed exclusive δ-opioid receptor mediated I/R injury reduction in rats utilizing a pure δ-agonist, also noting the effect was negated with the administration of opioid antagonists such as naltrexone [10]. The effects of morphine would be further corroborated by Lu et al. and Murphy et al. [11-13]. Fentanyl and its interaction with the κ-opioid receptor was shown to have similar effects via Xu et al. and again via another κ-receptor agonist in Zhang et al. [14, 15]. The exact physiological mechanism of the cardioprotective effects of κ and δ-opioid receptors is poorly understood.

Given the above, rapidly-induced opioid withdrawal may create an environment in which cardiac risk is elevated. As we have observed, opioids acting on κ and δ-opioid receptors convey a cardioprotective effect via a poorly understood mechanism. One can postulate that the sudden decrease in opioid receptor activity seen in our patient, especially given her elevated cardiac risk, would cause the opposite effect. However, this mechanism is merely conjecture and merits further investigation.

## Conclusions

Opioids exhibit a complex relationship with cardiac function that can be observed both via their direct effects on κ and δ-opioid receptors as well as indirect effects on myocardial oxygen consumption and afterload. The NSTEMI observed in our patient was likely due to increased myocardial strain and subsequent oxygen demand in the setting of rapidly-precipitated opioid withdrawal but it may have also been exacerbated by the sudden lack of cardioprotective effect conveyed by some opioids. It is likely that the morbidity caused could have been avoided with careful patient counseling as to how and when to take her medications. Practitioners should be aware of the possible cardiac side effects of opioid withdrawal, especially in those with elevated ASCVD risk. Meticulous efforts should be made to instruct patients as to the proper dosing schedule when initiating opioid therapy, as well as when initiating MOUD/OAT. It is our hope that these measures will both protect and educate this susceptible patient population.

## References

[1] Saunders JB, Conigrave KM, Latt NC (2016). Addiction Medicine. Addiction Medicine.

[2] Masoomi M, Zare J, Nasri H, Mirzazadeh A, Sheikhvatan M (2011). Abrupt opium discontinuation has no significant triggering effect on acute myocardial infarction. J Cardiovasc Med.

[3] Dadpour B, Gholoobi A, Tajoddini S, Habibi A (2017). Acute myocardial infarction following naltrexone consumption; a case report. Emerg (Tehran).

[4] (2020). Opioid antagonist. https://www.accessdata.fda.gov/drugsatfda_docs/label/2013/018932s017lbl.pdf.

[5] Smith HS (2013). Opioid Therapy in the 21st Century. https://www.ovid.com/product-details.6083.html.

[6] Paronis CA, Bergman J (2011). Buprenorphine and opioid antagonism, tolerance, and naltrexone-precipitated withdrawal. J Pharmacol Exp Ther.

[7] Schultz JE, Gross GJ (2001). Opioids and cardioprotection. Pharmacol Thera.

[8] Rong F, Peng Z, Ming-Xiang Y (2009). Myocardial apoptosis and infarction after ischemia/reperfusion are attenuated by kappa-opioid receptor agonist. Arch Med Res.

[9] Gross ER, Hsu AK, Gross GJ (2009). Acute methadone treatment reduces myocardial infarct size via the delta-opioid receptor in rats during reperfusion. Anesth Analg.

[10] Maslov LN, Barzakh EI, Krylatov AV (2010). Opioid peptide deltorphin II simulates the cardioprotective effect of ischemic preconditioning: role of δ2-opioid receptors, protein kinase C, and KATP channels. Bull Exp Biol Med.

[11] Lu Y, Dong C, Yu J, Li L (2011). Role of central and peripheral opioid receptors in the cardioprotection of intravenous morphine preconditioning. Irish J Med Sci.

[12] Murphy GS, Szokol JW, Marymont JH, Avram MJ, Vender JS (2006). Opioids and cardioprotection: the impact of morphine and fentanyl on recovery of ventricular function after cardiopulmonary bypass. J Cardiothorac Vasc Anesth.

[13] Murphy GS, Szokol JW, Marymont JH, Avram MJ, Vender JS (2007). The effects of morphine and fentanyl on the inflammatory response to cardiopulmonary bypass in patients undergoing elective coronary artery bypass graft surgery. Anesth Analg.

[14] Xu Y-C, Li R-P, Xue F-S (2015). κ-Opioid receptors are involved in enhanced cardioprotection by combined fentanyl and limb remote ischemic postconditioning. J Anesth.

[15] Zhang S, Zhou Y, Zhao L (2018). κ-opioid receptor activation protects against myocardial ischemia-reperfusion injury via AMPK/Akt/eNOS signaling activation. Eur J Pharmacol.

